# Evidence for the Use of Acupuncture in Treating Parkinson's Disease: Update of Information From the Past 5 Years, a Mini Review of the Literature

**DOI:** 10.3389/fneur.2018.00596

**Published:** 2018-07-25

**Authors:** Fan Jiang, Tiansong Yang, Hongna Yin, Yuhuai Guo, Hiroki Namba, Zhongren Sun, Tetsuya Asakawa

**Affiliations:** ^1^Heilongjiang University of Chinese Medicine, Harbin, China; ^2^First Affiliated Hospital, Heilongjiang University of Chinese Medicine, Harbin, China; ^3^Department of Neurosurgery, Hamamatsu University School of Medicine, Handayama, Hamamatsu-city, Japan; ^4^Research Base of Traditional Chinese Medicine Syndrome, Fujian University of Traditional Chinese Medicine, Fuzhou, China

**Keywords:** Parkinson's disease, acupuncture, electroacupuncture, non-motor symptoms, efficacy/safety, behavioral assessment

## Abstract

Acupuncture is an alternative therapy for Parkinson's disease (PD), but its efficacy and safety are controversial. Our previous study, which reviewed the literature from 1974 to 2012, could not find enough evidence from rigorously designed randomized, controlled trials (RCTs) to make a conclusion about the efficacy of acupuncture. Recently, more RCTs and meta-analyses have been conducted to evaluate the efficacy of acupuncture. The aim of our current study is to provide updated information in brief on this topic. In this study, we analyzed and summarized seven RCTs and four meta-analyses. Although all included studies were not of high quality, we found that there has been a tremendous progress in acupuncture research in treating Parkinson's disease (PD) during the past 5 years, based on our experience and insights into the behavioral assessments of PD. First, the numbers of RCTs and meta-analyses based on RCTs are increasing. Second, non-motor symptoms are increasingly emphasized. Third, objective behavioral assessment tools are being employed. Although recent studies can provide limited evidence for the efficacy of acupuncture, we make the following recommendations for the future investigation: First, large, multicenter, well-designed RCTs should be organized for evaluation of the efficacy of acupuncture. Second, objective assessments using novel computerized technologies should be considered. Third, target symptoms should be selected and evaluated instead of only performing global evaluations. Fourth, attention should be paid to the efficacy of scalp acupuncture. Fifth, the safety of acupuncture should be evaluated and reported.

## Introduction

Parkinson's disease (PD), the second most common neurodegenerative disorder, is a major health concern for elderly people. Classical therapies (CTs), such as dopaminergic medication and deep brain stimulation, are far from satisfactory. Before next-generation treatments, such as stem cell and genetic therapy, can be clinically applied, many alternatives are being considered as adjuvant therapies to improve the outcome of patients. Acupuncture, based on the theory of traditional Chinese medicine, has been used to treat PD, especially in the East Asian countries of China, Japan, Korea, and Singapore. Although the action mechanisms of acupuncture for treating PD remain unclear, the therapy may provide relief by affecting the progress of neuron degeneration, improving the dopaminergic system, improving the motor control network, and relieving oxidative stress (1). The included acupuncture studies involved two types of acupunctural methods: classical and electroacupuncture. While classical acupuncture warrants increased experience and skill of a therapist, electroacupuncture has objective parameters that can be easily achieved by a beginner. Therefore, electroacupuncture will be the future of acupuncture. In this study, the term “acupuncture” implies classical and electroacupuncture.

The efficacy of acupuncture for PD is controversial. At present there are no authoritative reports of the efficacy and safety of acupuncture for Parkinson's disease. Our 2013 review (1) covered almost all the available literature on this topic from 1974 to 2012 since the first report of the use of acupuncture in the central nervous system (2). This review could not find enough evidence from rigorously designed, randomized, controlled trial (RCTs) to make a conclusion about the efficacy of acupuncture. Many trials were not convincing because of flaws in the methodology (1, 3). We therefore wrote another review aiming to briefly introduce improved methodology to studies of acupuncture (3). In recent years, with progress in evidence-based medicine and clinical epidemiology, more RCTs and meta-analyses have been conducted to evaluate the efficacy of acupuncture. Moreover, non-motor symptoms of PD have been increasingly emphasized by clinicians. Many subjective assessment tools have been used for evaluation of PD symptoms (4, 5). It is imperative to update the information on the efficacy of acupuncture taking account of these advances in acupuncture studies. Although several systematic reviews have investigated the efficacy of acupuncture, we performed a newer and more comprehensive mini-review [the continuation of our previous study (1)] of these previous studies, as well as the newest RCTs. This review aims to provide updated, brief “take-home messages” about the efficacy of acupuncture in patients with PD.

## Materials and methods

**Figure 1:**
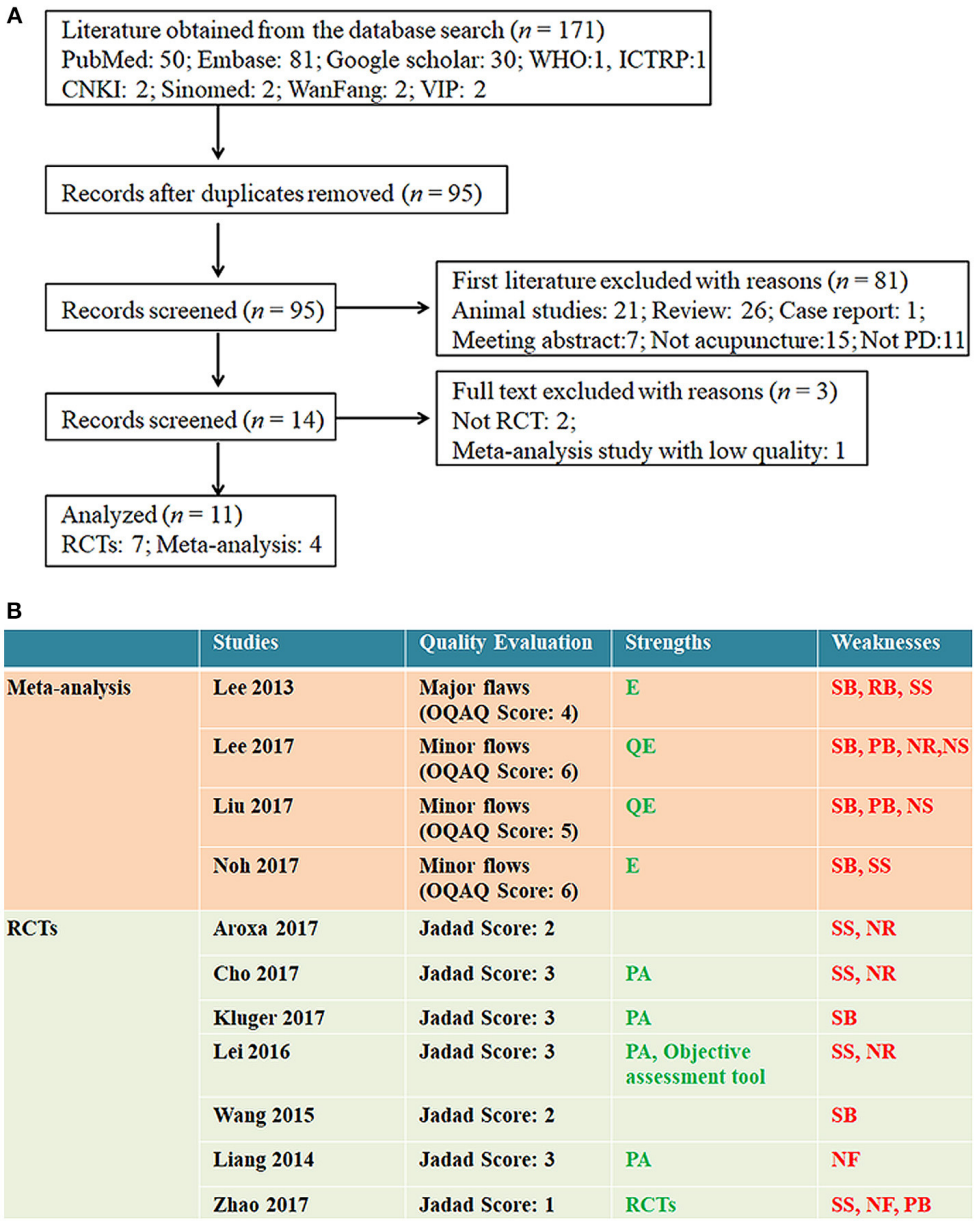
Information of the included literatures. **(A)** Flow chart of the literature selection protocol. PD, Parkinson's disease; RCT, randomized, controlled trial; WHO, World Health Organization. **(B)** Quality evaluation of included studies. OQAQ, Overview Quality Assessment Questionnaire. Strengths: E, Good searching strategy including enough RCTS; QE, Qualify of involved RCTs was evaluated; PA, Placebo acupuncture. Weaknesses: SB, Selection bias; RB, reporting bias; PB, Publication bias; SS, Small samples; NR, Adverse events unreported; NS, No sensitivity analysis NF, No follow up.

We searched the literature from English (PubMed, EMBASE, and Google Scholar) and Chinese (CNKI, SinoMed, VIP, and Wanfang Data) databases. In order to balance sensitivity and specificity, we also searched related trials via the World Health Organization (WHO) trials portal (ICTRP). Documents from 2013 to 2017 were included. The search results are shown in Figure 1A. A total of 171 studies were found. Finally, 11 studies, including 4 meta-analysis studies and 7 RCTs, were approached for further analysis (Figure 1A).

## Results

### Characteristics of included literature

A total of 11 studies were included, comprising 4 meta-analysis studies and 7 RCTs. The characteristics of the included studies are shown in Table 1. Subjective behavioral assessments employed in this study include the Unified Parkinson's Disease Rating Scale (UPDRS) (6–9, 11–16), the Webster Scale (6, 7, 9, 16), the Tension Assessment Scale (TAS) (9), the Parkinson's Disease Sleep Scale (PDSS) (10, 12, 15), the Postural Instability Gait Disorder (PIGD) (11), the Parkinson's Disease Quality of Life Questionnaire (PDQL) (11), the Beck Depression Inventory (BDI) (11), the Modified Fatigue Impact Scale (MFIS) (12), the 39-Item Parkinson's Disease Questionnaire (PDQ-39) (12, 15), and the Pittsburgh Sleep Quality Index (PSQI) (14). One RCT used gait speed as an objective assessment (13). Besides motor symptoms, non-motor symptoms, such as sleep (12, 14, 15), quality of life (QOL) (12, 15), and fatigue (12), were also evaluated. Objective evaluation and evaluation of non-motor symptoms are increasingly emphasized for evaluation of the efficacy and safety of acupuncture for PD. No serious adverse events were reported.

**Table 1:** Efficacy and safety in the current acupuncture study.

**Study**	**Experimental design treatment vs. control**	**Number of participants**	**Acupoints involved**	**Course**	**Assessments**	**Adverse events**	**Results**
**META-ANALYSIS OF ACUPUNCTURE FOR PD PATIENTS**							
Lee et al. (6)	A1. SA+CT vs. B1. CT alone A2. SA vs. B2. CT alone	184 participants including 4 RCTs	MS (4, 6, 8, 9, 14), MCA, CTCA, FMSA	>5 weeks average	UPDRS total score 2. Webster Scale	No serious adverse events reported	A1 > B1, *P* = 0.01, *I^2^* = 0%^*^ 2. A2 > B2, *P* = 0.30, *I^2^* = 84%
Lee et al. (7)	A1. Acup + CT vs. B1. CT alone A2. Acup vs. B2. No treatment A3. Acup vs. B3. CT alone	1616 participants including 25 RCTs	Taichong (LR-3), Baihui (GV-20), Yanglingquan (GB-34), Fengchi (GB-20), Hegu (LI-4), Sishencong (EX-HN1), Quchi (LI-11) (Freq ≥ 7)	> 6 weeks average	UPDRS I UPDRS II UPDRS III UPDRS IV UPDRS total scores Webster Scale 7.Total efficacy	Unreported	1: A1 > B1, *P* = 0.23, *I^2^* = 0% 2: A1 > B1, *P* < 0.001, *I^2^* = 0%^**^ 3. A1 > B1, *P* < 0.001, *I^2^* = 0%^**^ 4. A1 > B1, *P* = 0.17, *I^2^* = 93% 5. A1 > B1, *P* < 0.001, *I^2^* = 0%^**^ 6. A2 > B2, *P* < 0.001, *I^2^* = 0%^**^ A3 > B3, *P* < 0.001, *I^2^* = 0%^**^ A1 > B1, *P* < 0.001, *I^2^* = 93%^**^ 7. A3 > B3, *P* = 0.06, *I^2^* = 0% A1 > B1, *P* < 0.001, *I^2^* = 73%^**^
Liu et al. (8)	A. Acup + Madopar vs. B. Madopar alone	831 participants including 11 RCTs	Unreported	Unreported	Total efficacy UPDRS I UPDRS II UPDRS III UPDRS IV 6. UPDRS total scores	No serious adverse events reported	1: A > B, *P* < 0.001, RR = 1.28^**^ 2: A > B, *P* = 0.06, *I^2^* = 47% 3: A > B, *P* = 0.006, *I^2^* = 82%^**^ 4: A > B, *P* = 0.17, *I^2^* = 95% 5: A > B, *P* = 0.30, *I^2^* = 96% 6: A > B, *P* = 0.002, *I^2^* = 77%^**^
Noh et al. (9)	A1. Acup+CT vs. B1. CT alone A2. EA+CT vs.B2. CT alone A3. EA + Acup + CT vs.B3. CT alone A4. Acup vs.B4. Sham Acup	2625 participants including 42 RCTs	Taichong (LR-3), Fengchi (GB-20), Yanglingquan (GB-34), Hegu (LI-4), Baihui (GV-20), Zusanli (ST-36), Sishencong (EX-HN1)(Freq≥10)	> 4 weeks average	UPDRS total score Webster Scale 3. Tension Assessment Scale	No serious adverse events reported	1: A1 > B1, *P* < 0.00001, *I^2^* = 81%^**^ A2 > B2, *P* = 0.0006, *I^2^* = 46%^**^ A3 > B3, *P* = 0.003, *I^2^* = 0%^**^ A4 > B4, *P* = 0.59, *I^2^* = 0% 2: A1 > B1, *P* = 0.006, *I^2^* = 81%^**^ A2 > B2, *P* = 0.41, *I^2^* = 97% 3: A2 > B2, *P* = 0.08, *I^2^* = 38%
**RCTS OF ACUPUNCTURE FOR PD PATIENTS**							
Aroxa et al. (10)	A. Acup + drug vs. B. drug alone	22 participants	Taichong (LR-3), Sanyinjiao (SP-6), Hegu (LI-4), Waiguan (TE-5), Shenmen (HT-7), Neiguan (PC-6), Quchi (LI-11), Fengchi (GB-20)	8 weeks	PDSS score	Unreported	A > B, *P* = 0.66
Cho et al. (11)	Acup + BVA vs. Sham + vehicle vs. C. CT	63 participants	Fengchi (GB-20), Quchi (LI-11), Yanglingquan (GB-34), Zusanli (ST-36), Taichong (LR-3)	12 weeks	UPDRS II UPDRS III UPDRS II + III PIGD PDQL 6. BDI	No serious adverse events reported	1: A > C, *P* = 0.001^**^ A > B, *P* = 0.257 2: A > C, *P* = 0.008^**^ A > B, *P* = 0.793 3: A > C, *P* = 0.001^**^ A > B, *P* = 0.444 4: A > C, *P* = 0.001^**^ A > B, *P* = 0.244
Kluger et al. (12)	A. Acup vs. B. Sham Acup	89 participants	Baihui (GV-20), Shenting (GV-24), Qihai (CV-6), Shousanli (LI-10), Shenmen (HT-7), Zusanli (ST-36), Sanyinjiao (SP-6)	6 weeks	MFIS: Total MFIS: Physical MFIS: Cognitive MFIS: Psychosocial UPDRS III PDQ-39 Total 7. PDSS	No serious adverse events reported	1: A > B, *P* = 0.4388 2: A > B, *P* = 0.1881 3: A > B, *P* = 0.9222 4: A > B, *P* = 0.5638 5: A > B, *P* = 0.9343
Lei et al. (13)	A. EA vs. B. sham Acup	15 participants	FMSA, BA, Baihui (GV-20), Dazhui (GV-14), Hegu (LI-4), Zusanli (ST-36), Yanglingquan (GB-34), Weizhong (BL-40), Sanyinjiao (SP-6), Taixi (KI-3), Taichong (LR-3)	3 weeks	Gait-Speed UPDRS I UPDRS II 4. UPDRS III	No serious adverse events reported	1: A > B, *P* = 0.001^**^ 2: A > B, *P* = 0.005^**^ 3: A > B, *P* = 0.02^*^ 4: A > B, *P* < 0.001^**^
Wang 2015 (14)	A. EA + drug vs. B. drug alone	50 participants	Fengchi (GB-20), Hegu (LI-4), Dazhui (GV-14), Fengfu (GV-16)	2 months	UPDRS III 2. PSQI	Unreported	1: A > B, *P* = 0.036^*^ 2: A > B, *P* = 0.034^*^
Liang and Chen (15)	A. Acup vs. B. drug	70 participants	Fengchi (GB-20), Wangu (GB-12), Tianzhu (BL-10), Yamen (GV-15)	6 months	PDQ-39 UPDRS II 3. PDSS	Unreported	1: A > B, *P* < 0.001^**^ 2: A > B, *P* = 0.041^*^ 3: A > B, *P* < 0.001^**^
Zhao 2017 (16)	A. Acup + drug vs. B. drug	108 participants	Taichong (LR-3), Fengchi (GB-20), Hegu (LI-4), Sishencong (EX-HN1)	3 months	UPDRS total scores 2. Webster Scale	No serious adverse events reported	1: A > B, *P* = 0.005^**^ 2: A > B, *P* = 0.001^**^

* p < 0.05;

** *p < 0.01*.

### Quality assessment of included studies

We assessed the quality of the included studies with a Jadad scale (for RCTs) (17) and an Overview Quality Assessment Questionnaire (OQAQ) (for meta-analysis) (18) (Figure 1B).

The included meta-analyses were of minor (three studies) and major (one study) flaws because of the possible selection bias and small sample size. Although Lee (6) and Lee (7) used the Physiotherapy Evidence Database scale and Cochrane risk of bias to perform a quality assessment, there was a possible publication and performance bias, and most (80%) of the included RCTs had a serious selection bias. In addition, follow-up and sensitivity analyses were available in this study. The limitations of the study Lee 2013 were that only four RCTs published in China were included and the small sample size in these RCTs hindered drawing a useful conclusion (6). Although RCTs included in the study by Liu were published in Chinese, these were not based on a double-blind design and reported no follow-ups. Of the 11 RCTs, nine had a serious selection bias and three reported data loss. Moreover, the sensitivity analysis was not performed (8). In Noh's study, all RCTs had a performance bias and 39 of 42 RCTs had a selection bias. In addition, most studies included had a small sample size, and only seven out of 42 RCTs reported a follow-up (9). Of note, some overlaps occurred among the four meta-analyses. For example, four RCTs by Lee (6) were included in Lee (7). Of note, two studies of Lee (6) were also included in Noh's study, and four RCTs were included in the studies by Lee (7), and Noh and Liu. In addition, Liu's study comprised four studies overlapping with Lee's study (7) and six with Noh's study. Furthermore, Lee's study (2017) had 18 RCTs overlapped with the Noh's study.

The primary limitations of the included RCTs were small sample size and unreported adverse events. One progress compared with previous acupuncture studies was that placebo acupuncture was performed in four studies and objective behavioral assessment tool was used in one study Figure 1B.

### Efficacy of acupuncture for global evaluation

For global evaluation, we used the total UPDRS score and the Webster Scale. UPDRS is the most widely used scale for evaluation of the PD symptoms. The classic UPDRS has six parts: UPDRS I- mentation, behavior and mood; Part II—activities of daily life (ADLs); Part III—motor evaluation; Part IV—complications of therapy; Part V—staging of severity of PD; and Part VI—Schwab and England ADL scale. This is a clinician-report scale. Most items have scores ranged from 0 (normal) to 4 (severest). The Webster Scale is another commonly used self-report scale, which is briefer than UPDRS. It has 10 items (bradykinesia of hands, rigidity, posture, upper extremity swing, gait, no detectable tremor, tremor, seborrhea, speech, and self-care). The scores range from 0 (normal) to 3 (severest) (5). We found that all the measurements using the total UPDRS score showed good efficacy of acupuncture, regardless of whether the protocol acupuncture + classical treatment vs. classical treatment or acupuncture vs. classical treatment was used. A meta-analysis by Lee and Lim analyzed seven RCTs with 425 participants and found that the protocol acupuncture + classical treatment showed superior efficacy to classical treatment (weighted mean difference [WMD] = −10.73; 95% CI, −8.38 to−13.07; *P* < 0.001; *I*^2^ = 0%) (7). These results are similar to those of Liu et al. from 11 RCTs with 831 participants [standardized mean difference [SMD] = −1.15; 95% CI, −1.63 to −0.67; *P* < 0.001] (8), of Noh from five RCTs with 407 participants (WMD = −10.48; 95% CI, −13.61 to −7.34; *P* < 0.00001, *I*^2^ = 47%) (9), and of Lee from two RCTs with 60 participants (WMD = −3.94; 95% CI, −6.05 to −1.84; *P* = 0.01; *I*^2^ = 0%) (6).

A study by Lee et al. also employed the Webster Scale for global evaluation. The study found no significant differences between patients treated by acupuncture and controls (three RCTs with 154 participants; WMD = 1.29; 95% CI, 0.79 to 2.12; P = 0.30, *I*^2^ = 84%). However, the authors commented that the quality of the studies was low and the results were not convincing (6).

Only one recent RCT of 108 PD patients in China found efficacy of acupuncture + pramipexole + L-dopa vs. L-dopa. However, acupuncture + pramipexole + L-dopa had significantly better efficacy than L-dopa alone. The poor experimental design of this study could not distinguish whether the efficacy was from acupuncture, pramipexole, or both (16).

### Efficacy of acupuncture for motor symptoms

UPDRS III is the most commonly used score for measuring motor performance. An RCT with 15 participants conducted by Lei et al. evaluated improvement in motor performance due to electroacupuncture in 15 participants (13). They found that electroacupuncture significantly decreased UPDRS scores and improved gait speed. To the best of our knowledge, this is the first experiment using an objective task for evaluating the effects of acupuncture, which may provide rigorous evidence for evaluation of the efficacy of acupuncture. These results were in accordance with those of other studies by Cho et al. (one RCT with 63 participants) (11) and Lee et al. (a meta-analysis with five RCTs and 366 participants) (7). Only one study found no efficacy. A meta-analysis by Liu et al. that analyzed two RCTs with 240 participants did not found any efficacy of acupuncture in improving UPDRS scores (SMD = −0.93; 95% CI, −2.28 to 0.41; *P* = 0.17; *I*^2^ = 95%). Their explanation was that the high heterogeneity influenced the reliability of the results (8).

The Webster Scale is another subjective tool for assessment of motor performance. Only one poorly designed RCT with 108 participants evaluated the efficacy of acupuncture by this scale. The authors suggested that reduction of the Webster Scale score was significantly higher in the acupuncture group (16). Several meta-analyses, including those of Lee et al. (6) [three RCTs with 154 participants; WMD = −1.29; 95% CI,−0.79 to−2.12; *P* = 0.30; *I*^2^ = 84%] (6), Noh et al. (WMD = −1.99; 95% CI, −3.43 to −0.56; *P* = 0.006; *I*^2^ = 81%) (9), and Lee and Lim (7), came to the same conclusion that acupuncture has good efficacy for improving the motor symptoms as assessed by the Webster Scale. Lee and Lim found that acupuncture had good efficacy in comparison with no treatment (two studies with 74 participants; WMD = −7.36; 95% CI, −5.58 to −9.14; *P* < 0.001; *I*^2^ = 0%) and classical treatment (two studies with 260 participants; WMD = −3.08; 95% CI,−2.81 to −3.35; *P* < 0.001; *I*^2^ = 0%). They also found that acupuncture + classical treatment was better than classical treatment (four studies with 208 participants; WMD = -3.78; 95% CI,−2.17 to−5.40; *P* < 0.001; *I*^2^ = 93%) (7).

### Efficacy of acupuncture for non-motor symptoms

#### Tension/stress

The study of Noh et al. also investigated the efficacy of acupuncture for tension related to PD. They analyzed two RCTs with 121 participants and used the TAS to evaluate the efficacy of electroacupuncture to treat tension. They found that electroacupuncture + classical treatment had better efficacy than classical treatment alone (WMD = −1.85; 95% CI,−3.91 to−0.20; *P* = 0.08; *I*^2^ = 38%) (9). Although this is a stirring result, the limited number of RCTs (*n* = 2) and the small sample size (*n* = 121) limit the strength of the evidence.

#### Fatigue

Kluger et al. employed MFIS in an RCT to evaluate fatigue symptoms related to PD (12). They recorded MFIS for 6 weeks in 89 participants and found that acupuncture and sham acupuncture both had satisfactory efficacy (*p* < 0.0001). However, there was no evidence that acupuncture was superior to sham treatment (*p* < 0.34). In conclusion, acupuncture is good for relief from fatigue, regardless of it being PD-related or not. However, limitations of the experimental design, such as the lack of a wait-list control arm and potential selection bias (participants were highly educated), could have affected the reliability of this study. More rigorously designed RCTs are needed (19).

#### PD-related sleepiness

Three RCTs evaluated the efficacy of acupuncture for PD-related sleepiness. Aroxa et al. used the PDSS to compare the efficacy of acupuncture + drug with that of classical treatment alone in 22 participants. Although acupuncture produced a significant improvement, there was no evidence that acupuncture + drug was superior to drug alone (10). However, an RCT by Liang and Chen with 70 participants used PDSS and found that acupuncture was more efficacious than drug alone (15). Wang et al. used the PSQI to evaluate the efficacy of electroacupuncture for PD-related sleepiness in 50 participants. They found that electroacupuncture + drug significantly improved PSQI scores, whereas drug alone did not (14). The three RCTs revealed contradictory results. While one RCT (*n* = 22) denied any efficacy, the other two (*n* = 70 and 50) confirmed the efficacy. Hence, further well-designed, extensive studies are warranted for rigorous validation.

#### PD-related psychiatric symptoms

Based on the UPDRS I Scale (for examination of mentation, behavior, and mood), the results of a recent RCT were different in two meta-analysis studies. The RCT, with 15 participants, found that the acupuncture group achieved a significant improvement (*p* < 0.01), whereas the sham group did not (*p* = 0.21) (13). However, the meta-analysis studies (7, 8) found no significant improvement with acupuncture. Liu examined 240 patients in two trials and did not report any significant change in UPDRS I (SMD = −0.37; 95% CI: −0.77 to 0.02; *P* = 0.06) (8), Lee analyzed two studies (one was overlapped with Liu's study) and reported the same result (weighted mean difference = 0.27; 95% CI: 0.17–0.72; *P* = 0.23; *I*^2^ = 0%; *n* = 228) (7). However, the results were debatable. As the sample size in the RCT (*n* = 15) was too small, the efficacy of acupuncture in treating PD-related psychiatric symptoms warrants further validation by large, multicenter, well-designed RCTs.

#### QOL

Three RCTs using the UPDRS Scale found that acupuncture had significant efficacy for improving QOL in PD patients (11, 13, 15). Using a PDQ-39 questionnaire, Kluger et al. reported the scores of acupuncture in weeks 1 and 6 as 27.4 ± 10.0 and 21.6 ± 12.2, respectively, confirming a significant amelioration in PD symptoms (12). Lei's study mentioned that the QOL can be improved by improving the hypokinetic rigid gait of PD (13). A Chinese study by Liang and Chen reported PDQ-39 scores of 20.41 ± 11.64 and 27.48 ± 8.69 in the acupuncture and control groups, respectively. This difference was significant. Furthermore, all five items of the QOL in PDQ-39 were significantly improved in the acupuncture group (15).

## Discussion

As a continuation of our previous studies (1, 3), the present study indicated that several studies, including RCTs and meta-analysis studies, provided limited evidence for the efficacy of acupuncture to treat PD, including motor and non-motor symptoms. There is no authoritative evidence from rigorously designed, large-scale, multicenter RCTs. However, compared with the previous study (1) 5 years ago which we could not make a conclusion regarding the efficacy of acupuncture, the situation has been improved.

### Improvements/characters in acupuncture studies during the past 5 years

#### RCTs and meta-analysis studies based on RCTs are increasing

More rigorously designed RCTs and meta-analyses following PRISMA rules were used to evaluate the efficacy of acupuncture. This will provide more convincing evidence for the efficacy of acupuncture. Increasing numbers of acupuncture investigators have accepted the principles of evidence-based medicine and recognized that only well-designed RCTs can provide powerful and convincing evidence for the efficacy of acupuncture, which is the only way for acupuncture to step into mainstream medical academia. In the present study, seven recent RCTs and four meta-analysis studies evaluated the efficacy of acupuncture. Although some studies did not reach a satisfactory quality (Figure 1B), the data obtained from RCTs cannot be ignored.

#### Non-motor symptoms are increasingly emphasized

In addition to the motor symptoms of PD, more clinicians are paying attention to the non-motor symptoms (4, 5). We found several studies that evaluated the efficacy of acupuncture for non-motor symptoms. Noh et al. found that acupuncture was effective against PD-related stress (9); Kluger reported that acupuncture could relieve PD-related fatigue (19); studies by Liang et al. (15) and Wang et al. (14) showed that acupuncture could relieve PD-related sleepiness; and QOL in PD patients could be improved by acupuncture (11, 13, 15). The non-motor symptoms of PD are quite complex. More investigations can be expected to verify the efficacy of acupuncture for various non-motor symptoms in PD.

#### Objective behavioral assessment tools are employed in acupuncture studies

Behavioral assessments play a crucial role in evaluation of PD symptoms. Selection of different assessment tools may lead to different results. Currently, most acupuncture studies have employed subjective scores such as UPDRS. However, this may cause observation bias. In the present study, we found that one study used an objective task (gait speed) to evaluate the efficacy of acupuncture (13). Because of the peculiarity of acupuncture, it is difficult to use a double-blind design in the studies (1). Objective indexes in the experimental design are required to avoid observation bias (3). On the other hand, the principles of objectification, multipurpose, and simplification (OMS) (4, 5) have been the tendency of behavioral assessments in PD. We believe objective assessment tools will be developed and employed in future studies.

### Recommendations for future studies

Based on the recent acupuncture studies summarized here, we make several recommendations for future studies.

1. The quality assessments (Figure 1B) suggested that some studies were of low quality and some items (such as PD-related psychiatric symptoms and sleepiness) provided inconsistent results in different studies. Thus, it is time to organize large, multicenter, well-designed RCTs to evaluate the efficacy of acupuncture. Although many RCTs have provided evidence for the efficacy of acupuncture, flaws of methodology (small sample size, statistical flaws, etc.) have reduced the value of the evidence. Acupuncture needs authoritative evidence to step into the mainstream of medical academia. Once acupuncture has been authoritatively proved to be efficacious and accepted by clinicians, it should be considered as a method of “peripheral stimulation” to treat PD (20). Peripheral stimulation has no surgical risks, which will greatly benefit PD patients in the world.

2. Objective assessments using novel computerized technologies, such as wearable devices, virtual reality, and augmented reality technologies and robot assistant technology, which can realize real-time, programmable, and safe measurements of the motor fluctuations in PD, should be developed and used in future acupuncture studies to provide more powerful evidence for the efficacy of acupuncture, since it may bring great revolutionary in the behavioral assessments in PD (5).

3. Because of the complicated nature of PD symptoms, we suppose that different acupuncture parameters (acupoints, duration time, current intensity, and frequency, etc.) may correspond to different symptoms. Future investigations should evaluate the efficacy of acupuncture according to the target symptoms observed, instead of only performing global evaluations.

4. The safety of acupuncture should be evaluated. Recent studies have emphasized the evaluation of efficacy. In this study, none of the enrolled studies reported serious adverse events. However, to our knowledge, acupuncture is not totally harmless. Adverse events such as stabbing pain, hematoma, and bleeding have been reported (1). Perhaps, insufficient reporting of adverse events could decrease the reliability of studies; albeit some scientists believe that the safety of acupuncture is not a problem as evidenced by the lack of adverse events, the health risks cited are basically similar to venipuncture.

## Concluding remarks

In the past 5 years, progress has been made in providing limited evidence for the efficacy of acupuncture in treating PD. However, there is still no authoritative evidence, which has prevented acupuncture from stepping into the mainstream of medicine. More innovations, including experimental design and assessment tools, are recommended for the future validation of acupuncture. Acupuncture research should also keep pace with mainstream PD research.

## Author contributions

TY, FJ, ZS, and TA got the original idea. TY, FJ, HY, HN, ZS, and TA searched for the literatures. TY and FJ performed the data analysis. TA wrote the draft. TY, FJ, HY, HN, ZS, and TA revised and approved the final manuscript. TA and ZS supervised the study.

### Conflict of interest statement

The authors declare that the research was conducted in the absence of any commercial or financial relationships that could be construed as a potential conflict of interest.

## Abbreviations

Acup: acupuncture
BA: balance area
BDI: Beck Depression Inventory
CT: classical treatment
CTCA: Chorea-tremor controlling area
EA: electroacupuncture
FMSA: foot motor sensory area
MCA: motor controlling area
MFIS: Modified Fatigue Impact Scale
PDQ-39: 39-Item Parkinson's Disease Questionnaire
PDQL: Parkinson's Disease Quality of Life Questionnaire
PDSS: Parkinson's Disease Sleep Scale
PIGD: Postural Instability and Gait Disturbance
PSQI: Pittsburgh Sleep Quality Index
RCT, randomized: controlled trial
TAS: Tension Assessment Scale
UPDRS: Unified Parkinson's Disease Rating Scale
UPDRS I, mentation: behavior and mood subscale of the Unified Parkinson's Disease Rating Scale
UPDRS II: activities of daily living subscale of the Unified Parkinson's Disease Rating Scale
UPDRS III: motor symptoms caused by Parkinson's disease subscale of the Unified Parkinson's Disease Rating Scale
UPDRS IV: complications of therapy subscale of the Unified Parkinson's Disease Rating Scale.
